# Resting-State EEG Microstates and Power Spectrum in Borderline Personality Disorder: A High-Density EEG Study

**DOI:** 10.1007/s10548-023-01005-3

**Published:** 2023-09-30

**Authors:** Marie-Pierre Deiber, Camille Piguet, Cristina Berchio, Christoph M. Michel, Nader Perroud, Tomas Ros

**Affiliations:** 1 Department of Psychiatry, University Hospitals of Geneva, Chemin du Petit-Bel-Air 2, 1226 Thônex, Geneva, Switzerland; 2 Department of Psychiatry, Faculty of Medicine, University of Geneva, Geneva, Switzerland; 3 Department of Pediatrics, University Hospitals of Geneva, Geneva, Switzerland; 4 Department of Pediatrics, University of Geneva, Geneva, Switzerland; 5 Functional Brain Mapping Laboratory, Department of Fundamental Neuroscience, University of Geneva, Geneva, Switzerland; 6 Center for Biomedical Imaging, CIBM, Lausanne, Switzerland; 7 Division of Psychiatric Specialties, Department of Psychiatry, University Hospitals of Geneva, Geneva, Switzerland; 8 Department of Neuroscience, University of Geneva, Geneva, Switzerland

**Keywords:** Borderline personality disorder (BPD), EEG, Resting-state, Microstates (MS), Alpha rhythm

## Abstract

Borderline personality disorder (BPD) is a debilitating psychiatric condition characterized by emotional dysregulation, unstable sense of self, and impulsive, potentially self-harming behavior. In order to provide new neurophysiological insights on BPD, we complemented resting-state EEG frequency spectrum analysis with EEG microstates (MS) analysis to capture the spatiotemporal dynamics of large-scale neural networks. High-density EEG was recorded at rest in 16 BPD patients and 16 age-matched neurotypical controls. The relative power spectrum and broadband MS spatiotemporal parameters were compared between groups and their inter-correlations were examined. Compared to controls, BPD patients showed similar global spectral power, but exploratory univariate analyses on single channels indicated reduced relative alpha power and enhanced relative delta power at parietal electrodes. In terms of EEG MS, BPD patients displayed similar MS topographies as controls, indicating comparable neural generators. However, the MS temporal dynamics were significantly altered in BPD patients, who demonstrated opposite prevalence of MS C (lower than controls) and MS E (higher than controls). Interestingly, MS C prevalence correlated positively with global alpha power and negatively with global delta power, while MS E did not correlate with any measures of spectral power. Taken together, these observations suggest that BPD patients exhibit a state of cortical hyperactivation, represented by decreased posterior alpha power, together with an elevated presence of MS E, consistent with symptoms of elevated arousal and/or vigilance. This is the first study to investigate resting-state MS patterns in BPD, with findings of elevated MS E and the suggestion of reduced posterior alpha power indicating a disorder-specific neurophysiological signature previously unreported in a psychiatric population.

**Supplementary Information:**

The online version contains supplementary material available at 10.1007/s10548-023-01005-3.

## Introduction

Borderline personality disorder (BPD) is a mental disorder with a prevalence of around 1.6% in the general population (Kulacaoglu and Kose 2018). It is characterized by marked psychological dysregulation in several domains, including affect, sense of self, interpersonal relationships, as well as enhanced hostility, impulsivity and risk taking (Gunderson et al. 2018; Kulacaoglu and Kose 2018; Lieb et al. 2004). BPD patients often present with several comorbidities, and the disorder represents a severe personal and societal burden. One of the ways to gain a better understanding of its underlying pathophysiology is to identify brain biomarkers of the disorder. The characterization of specific neurophysiological patterns associated with BPD could potentially assist the diagnosis and guide therapeutic intervention. While numerous fMRI studies have focused on brain activation differences during domain-sensitive tasks (Krause-Utz et al. 2014 for review), recent studies have started to examine brain activity during rest, by analyzing spontaneous neural activity and functional connectivity (Visintin et al. 2016 for review). In this context, fMRI signals have a relatively low temporal resolution of several seconds, and spontaneous brain networks can be better explored on sub-second temporal scales with EEG (Varela et al. 2001 for review). Recent EEG studies in BPD have reported increased prevalence of intermittent delta and theta oscillatory activities which are supportive of medical care consisting of anticonvulsive treatment (Tebartz van Elst et al., 2016). On the contrary, frontal alpha asymmetry, a functional biomarker of emotion and motivation processing (Smith et al. 2017), was not significantly different in BPD compared to controls, but the scores of alexithymia were negatively associated with right-frontal alpha activity (Flasbeck et al. 2017). There is some debate concerning brain arousal in BPD patients, where its dysregulation has been associated with higher levels of emotional instability (Hegerl and Hensch 2014). While lowered-EEG vigilance was initially reported (Hegerl et al. 2008), more recent evidence based on a computer-based scoring algorithm (VIGALL2.0, Sander et al. 2015) suggests elevated resting-state EEG vigilance in BPD patients (Kramer et al. 2019).

In addition to spectral power measures, the spatio-temporal dynamics of large-scale neural networks can be captured using EEG microstate analysis (Michel and Koenig 2018; Pascual-Marqui et al. 1995; Van de Ville et al. 2010). Frequently applied to spontaneous (i.e. resting-state) EEG, this analysis models the sequential occurrence of transient topographical patterns of electrical activity that are referred as “microstates” (MS). While historical analyses described 4 canonical MS topographies dominating resting-state activity (A, B, C, D, Britz et al. 2010; Michel and Koenig 2018), recent work has identified up to 7 main MS patterns within resting EEG (A, B, C, D, E, F, G, Brechet et al. 2019; Custo et al. 2017; Damborska et al. 2019b). A growing body of research indicates significant MS alterations in a variety of neuropsychiatric disorders, such as schizophrenia and depression (Khanna et al. 2015 for review). Our own group recently reported on abnormalities in EEG MS dynamics in adult attention-deficit hyperactivity disorder (ADHD), finding a positive association between microstate D duration and sleep disturbance (Ferat et al. 2021). In the current study, we applied resting-state MS analysis for the first time in patients with BPD, in the hope of providing new neurophysiological insights of this disorder and/or identifying potential targets for future treatments. Our objective was to examine both the EEG spectral power and microstate dynamics in parallel, in order to evaluate their respective contribution in characterizing the electrophysiological signature(s) of BPD. Using high-density EEG recordings (comprising 256 EEG-channels), we tested for statistical differences in spectral power and traditional MS measures between BPD patients (n = 16) and age-matched healthy controls (CTL, n = 16). Consequently, we examined the correlations between the most salient metrics derived from group-analyses with both approaches, as they may provide complementary information on the clinical aspects of the disorder.

## Materials and Methods

### Participants and Clinical Assessment

The present study analyzed a subgroup of participants enrolled in a larger neuroimaging study (Berchio et al. 2017; Murray et al. 2022). Sixteen BPD patients (15 female, mean age: 25.1 ± 5.6) diagnosed with BPD were recruited in a specialized unit of the Department of Psychiatry of the University Hospitals of Geneva. The majority of women in our sample reflects the worldwide overrepresentation of women diagnosed with BPD in specialized units (Sansone and Sansone 2011; Skodol and Bender 2003). A group of 16 age-matched healthy controls (10 female, mean age: 29.6 ± 13.5) were recruited in parallel through announcements in the population. There was no significant difference in gender between the two experimental groups (Table 1). Each participant filled their informed consent prior to the study, which was approved by the Research Ethic Committee of the Republic and Canton of Geneva (CER 13–081).

BPD diagnosis was assessed by the French version of the Structured Clinical Interview for DSM-IV Axis II Disorders BPD part (BPD severity index: M: 7.4, SD = 1.84). Depression was evaluated using the Montgomery-Åsberg Depression Rating Scale (MADRS, Montgomery and Åsberg 1979). In addition, participants completed several self-report questionnaires: the State-Trait Anger Expression Inventory (STAXI, Spielberger 2010), the State-Trait Anxiety Inventory (STAI, Spielberger et al. 1983), the Cognitive Emotion Regulation Questionnaire (CERQ, Jermann et al. 2006), the Affective Lability Scale (ALS, Harvey et al. 1989), the Ruminative Response Scale (RRS, Treynor et al. 2004), the Impulsive Behavior Scale (UPPS, Whiteside et al. 2005), and the Adult Self-Report Scale for ADHD (ASRS, Kessler et al. 2005). We computed sub-scores where relevant: adaptive and maladaptive emotion regulation strategies with the CERQ, rumination and reflection with the RRS, and 4 subscales of the UPPS. The groups differed in: anger and anxiety scores, cognitive non adaptative regulation, affective lability, rumination (except for reflection), impulsive behavior (except sensation seeking), inattention and impulsivity as assessed using ASRS (Table 1). Affective disorders, schizophrenia, and other comorbid conditions in BPD and CTL were assessed using the French version of the Diagnostic Interview for Genetic Studies (DIGS) (Preisig et al. 1999). In BPD patients, current comorbidities included: eating disorders (n = 1), post-traumatic stress disorder (n = 6), anxiety disorder (n = 3), attention-deficit-hyperactivity disorder (n = 2), and substance abuse (n = 5). Comorbidity information was missing for 3 patients. One patient was receiving psychotropic medication (quetiapine). Healthy control subjects had no history of psychiatric illness as assessed with the DIGS, and had no taken medication or substance by their own report.

**Table 1:** Demographics and self-report questionnaire scores of the two groups, mean (standard deviation). *MADRS* Montgomery-Åsberg Depression Rating Scale; *STAXI* State Anger Expression Inventory; *CERQ* Cognitive Emotion Regulation Questionnaire; *AdaptReg* adaptative regulation; *NonAdaptReg* non adaptative regulation; *ALS *Affective Lability Scale; *RRS* Ruminative Response Scale; *UPPS* Impulsive Behavior Scale; *ASRS* Adult Self Report Scale for ADHD; *STAI* State-Trait Anxiety Inventory. t value T-test for independent samples (gaussian data); *MW* *U* Non parametric Mann Whitney U test (non gaussian data)

Demographics, scores	BPD (n = 16)	Controls (n = 16)	t value *MW U*	p value
Age	25.06 (5.57)	29.56 (13.50)	*125.5*	0.926 ^NS^
Gender : female, n	15	10	*88.0*	0.138 ^NS^
Medicated^a^ (n)	1	0		
Education (years)	14.5 (2.83)	14.9 (3.18)	*113.5*	0.800 ^NS^
MADRS	9.85 (5.70)	1.20 (1.57)	*1*	< 0.001
STAXI-state	22.13 (7.65)	12.85 (2.67)	*19.5*	< 0.001
STAXI-trait	26.33 (6.58)	17.75 (4.58)	3.94	< 0.001
CERQ_AdaptReg	57.73 (14.78)	57.71 (10.99)	0.004	0.997 ^NS^
CERQ_NonAdaptReg	47.87 (11.49)	35.71 (8.00)	3.28	0.003
ALS total	1.83 (0.53)	0.34 (0.29)	*1*	< 0.001
RRS_short	26.43 (3.76)	20.14 (4.77)	3.87	0.001
RRS reflection	11.36 (1.91)	10.86 (2.82)	0.55	0.588 ^NS^
RRS brooding	15.07 (2.99)	9.29 (2.89)	5.19	< 0.001
UPPS urgency	37.43 (4.72)	23.86 (5.74)	6.84	< 0.001
UPPS lack premeditation	29.36 (5.00)	20.49 (4.94)	4.72	< 0.001
UPPS lack perseverance	25.14 (4.67)	16.71 (4.66)	4.78	< 0.001
UPPS sensation seeking	34.21 (6.86)	30.93 (8.86)	1.10	0.283 ^NS^
ASRS inattention	24.6 (6.27)	10.00 (4.85)	6.98	< 0.001
ASRS impulsivity	19.93 (6.68)	9.57 (5.77)	4.45	< 0.001
STAI-state	53.82 (13.26)	28.24 (4.64)	*4.50*	< 0.001
STAI-trait	58.45 (7.56)	33.93 (8.13)	8.27	< 0.001

^NS^: non significant

^a^Psychotropic medication only

### EEG Acquisition

EEG data were acquired with a 256-channel Electrical Geodesic Inc. system (Eugene, OR), with a sampling rate of 1000 Hz, and Cz as reference electrode. Electrode impedances were kept below 30 kOhms. Three minutes of resting-state with eyes closed were acquired at the beginning of an experimental procedure on face and gaze processing (Berchio et al. 2017).

### EEG Analysis

#### Pre-processing

Data were processed in MATLAB version 2021a with EEGLAB version 14 (The MathWorks, Inc.) (Delorme and Makeig 2004). Offline EEG data was firstly down-sampled to 250 Hz. Then, the following sequence of steps were performed in order to remove artifactual (i.e. non-cerebral) sources of electrical activity that may contaminate EEG recordings (Bailey et al. 2022). Firstly, EEG data was band-pass filtered at 1–80 Hz. Next, the Zapline method was used to remove the top 6 components around the 50 Hz main line frequency (de Cheveigne 2020). Then, we removed bad channels using EEGLAB’s PREP plugin (Bigdely-Shamlo et al. 2015) with default settings and spherically interpolated the rejected channels. After which, Infomax ICA was performed using the runica() function. We then rejected specific ICA components related to (i) eye blinks/movements using the EyeCatch algorithm default settings (Bigdely-Shamlo et al. 2013), and (ii) muscle artifacts flagged by ICLabel at > 50% probability (Pion-Tonachini et al. 2019). We then automatically removed additional low-frequency artifacts using wavelet ICA at threshold = 10 and wavelet level = 10 (Castellanos and Makarov 2006). Finally, remaining EEG artifacts were removed epoch-wise with a z-score based method using the FASTER plug-in (Nolan et al. 2010), rejecting 1-second epochs deviating by more than two standard deviations. Cleaned EEG data were visually inspected before and after automatic processing to verify the quality of deartifacting performed. All subjects’ clean EEG data exceeded 120 s. After deartifacting, the data were bandpass filtered between 1 and 30 Hz and re-referenced to a common average reference.

#### Power Spectrum Analysis

Absolute power spectral density was computed for frequencies ranging from 1 to 30 Hz using the Welch method, with a 2 s window effective size and no overlap. To obtain a relative metric usable for between-subject comparisons, all values were divided by the sum of the full spectrum (1–30 Hz). For further analysis, the obtained values were then added up within each studied frequency band: delta (2–4 Hz), theta (4–8 Hz), alpha (8–12 Hz), and beta (13–30 Hz).

#### Microstate Analysis

##### Estimation of Microstate Maps

The pipeline for estimating the MS topographies has been described elsewhere (Ferat et al. 2021). MS topographies were estimated separately for the BPD and control groups using Thomas Koenig’s Microstate toolbox v1 for EEGLAB. For each subject’s resting-state recording, 2000 global field power peaks were randomly selected and subjected to modified k-means clustering (polarity-independent) with 100 repetitions. MS maps (i.e., cluster centroids) were estimated for cluster numbers from k = 4 to k = 7, first at the subject level and then optimally reordered between subjects by minimizing the average spatial correlation across maps. Finally, the respective MS maps were averaged across all subjects within each group to obtain the aggregate map for each cluster. Based on the mean spatial correlation of each subject’s map with the group’s aggregate, we found that k = 5 provided the highest map reliability across subjects.

##### Backfitting

Thomas Koenig’s Microstate toolbox v1 for EEGLAB was used to backfit on the whole data the k = 5 global dominant topographies back to the original EEGs. The time points were assigned to cluster labels, or MS topographies, based on their highest absolute spatial correlation. Time points with a spatial correlation below the correlation threshold of r = 0.5 were labeled as non-assigned. To ensure temporal continuity, a smoothing window of seven samples (56.0 ms) was applied, and label sequences that did not reach a duration of 3 samples (24.0 ms) were split into two parts and relabeled based on the highest spatial correlation with their neighboring segment. Non-assigned time points being removed, none of the participants had z ≥ 3 for unlabeled time points and they were all included in further analysis. A label sequence was derived for each individual recording, and three spatiotemporal metrics were computed: occurrence (counts/second), mean duration (milliseconds), and time coverage (%), representing the number of times a microstate class recurred per second, the mean temporal duration without interruption, and the proportion of time during which a label was present in the recording, respectively.

### Statistical Analysis

#### EEG Spectral Power

Statistical analysis of EEG spectral power was carried out at the electrode level in the four frequency bands (as defined above: delta, theta, alpha, beta) using the Neurophysiological Biomarker Toolbox version 1 (NBT, http://www.nbtwiki.net/) in Matlab version 2021a (MathWorks Inc.). In absence of pre-established hypothesis, the two-sided test was used. P-values were estimated by simulated random sampling with 5000 replications. For univariate analyses at the single-channel level, no correction was applied for multiple comparisons, such that results remained exploratory in nature.

#### Microstate Measures

Group comparisons were conducted on the three MS spatiotemporal metrics using unpaired permutation test for equality of means (Ferat et al. 2021). In absence of pre-established hypothesis, the two-sided test was used. P-values were estimated by simulated random sampling with 5000 replications. Statistical results were corrected for multiple comparisons using False Discovery Rate (FDR), as proposed by Benjamini-Hochberg (Benjamini and Hochberg 1995). The significance threshold for all comparisons was set to alpha = 0.05. Cohen’s d (d) was used to report effect sizes as standardized difference of means.

#### Correlation Analyses

We additionally conducted exploratory Spearman correlation analyses (Spearman’s Rho) between spectral power and MS spatiotemporal metrics on the pooled BPD and control datasets in order to evaluate if these two categories of EEG features may target similar or distinct aspects of the disorder (Gordillo et al. 2023).

Furthermore, we explored the potential correlations of the affective lability score (ALS) with MS C and E measures from the pooled BPD and control group data (Spearman’s Rho). We selected the ALS as it is a specific hallmark of emotional dysregulation in BPD (Koenigsberg et al. 2002) validated in the French language and commonly used for case control clinical studies (Marwaha et al. 2018).

## Results

### Spectral Power Analysis

**Fig. 1:**
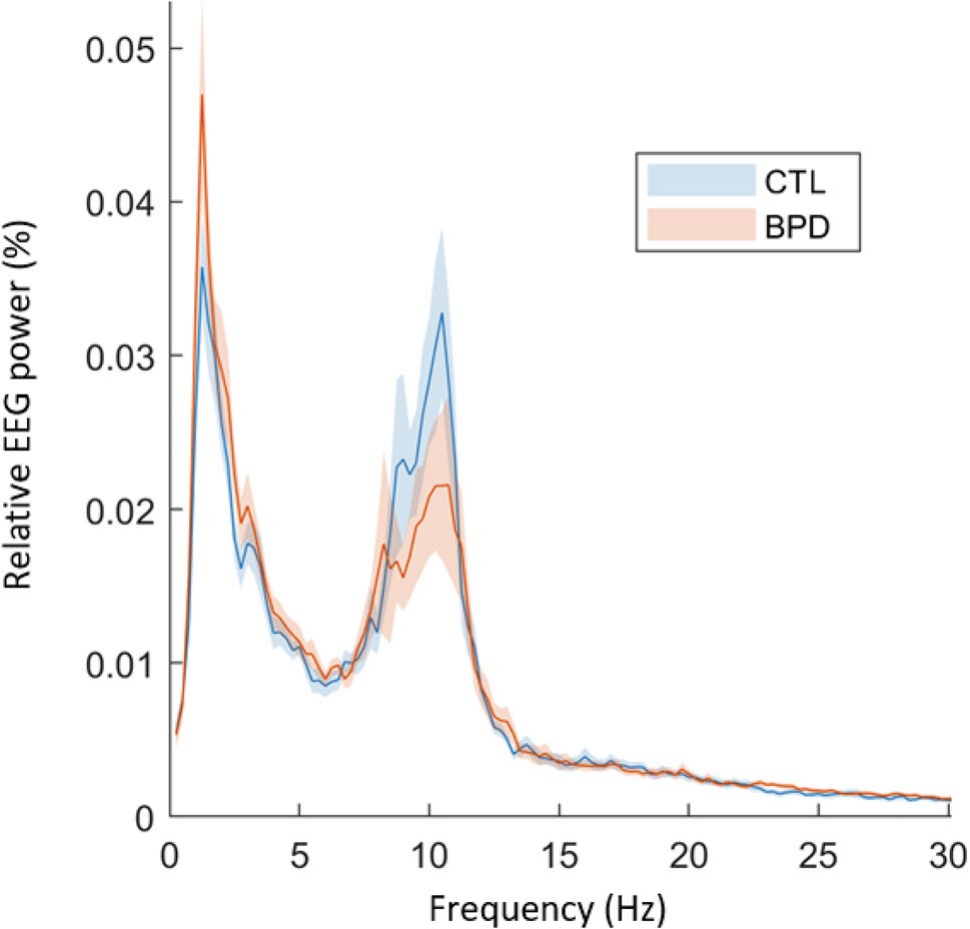
EEG relative power spectrum during eyes closed resting-state in BPD patients (orange) and control subjects (CTL, blue). Solid lines: mean relative power averaged over 256 electrodes. Shaded areas: 95% confidence interval

The EEG relative power spectrum is displayed in Fig. 1, showing reduced amplitude in the 8–12 Hz alpha frequency band and elevated amplitude in the 2–4 Hz delta frequency band in BPD compared to CTL.

**Fig. 2:**
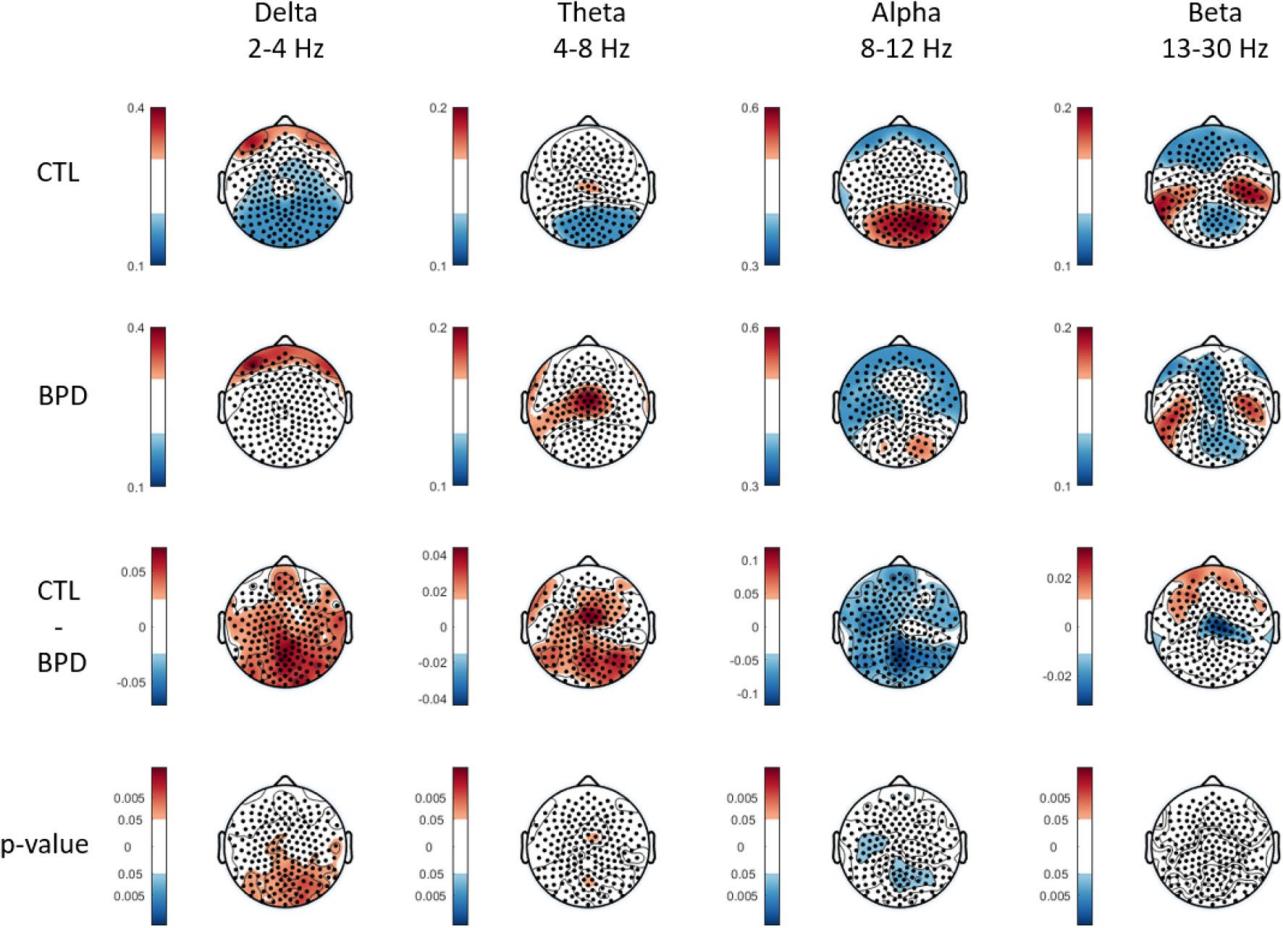
Topographic plots of relative delta, theta, alpha and beta power at eyes-closed rest for the CTL (first row) and BPD (second row) groups, and their mean difference (third row). The fourth row indicates significant channel-wise p-values following permutation tests (uncorrected, p < 0.05)

Here, no significant differences were detected between groups in global relative power (i.e. average of all channels) in any frequency bands. As shown in Fig. 2, univariate analyses on the single-channel level (p < 0.05 uncorrected) revealed significantly increased delta power (cluster maximum at O2 electrode: d = 0.93, p < 0.05 uncorrected) and decreased alpha power (cluster minimum at POz electrode: d = − 0.82, p < 0.05 uncorrected) in the posterior-midline region in BPD as compared to CTL (Fig. 2).

### MS Topographies

**Fig. 3:**
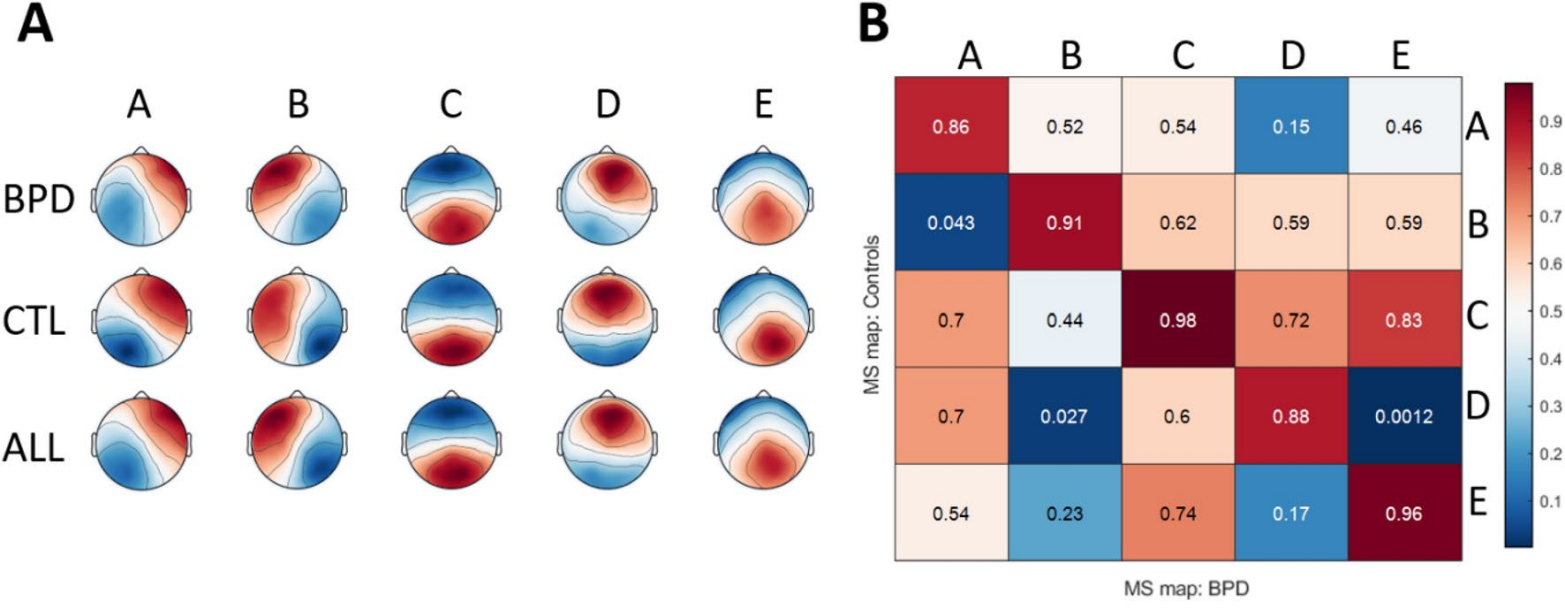
**A** The five EEG microstate topographies in adults with Borderline Personality Disorder (BPD, n = 16), control subjects (CTL, n = 16) and both groups averaged (ALL). **B** Spatial correlation coefficients of the five resting-state topographies between BPD and CTL

As shown in Fig. 3A, BPD and CTL groups both exhibited the 5 canonical MS topographies A, B, C, D, E (Michel and Koenig 2018), i.e., group maps with a right-left diagonal orientation (A), a left-right diagonal orientation (B), an antero-posterior orientation (C), a fronto-central maximum (D), and a parieto-central maximum (E). Spatial correlation analysis showed minor differences of MS maps between groups (minimum absolute correlation ≥ 0.86) (Fig. 3B). As the results from a k-means clustering on concatenated BPD and CTL data did not produce different MS topographies, we used the vector average between group maps for back-fitting and individual estimation of MS dynamics.

### MS Segmentation

**Fig. 4:**
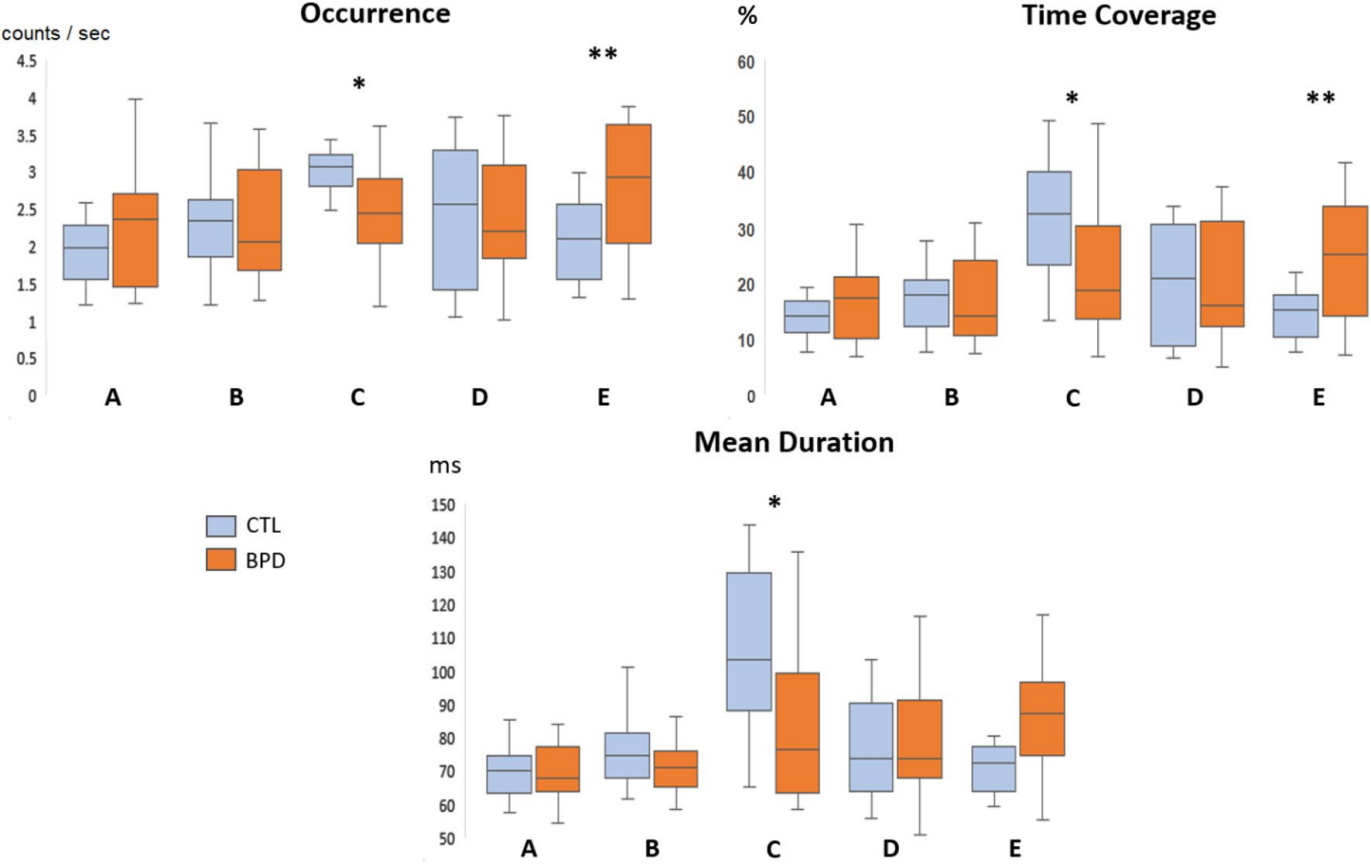
Between-group comparisons of the three temporal measures for each of the five microstates. *p < 0.05, **p < 0.01 based on permutation tests for mean difference, FDR corrected for multiple comparisons

Figure 4 shows the plots of the three MS spatiotemporal measures across groups. In the BPD compared to CTL group, MS C showed reduced occurrence (d = − 0.89, p = 0.010 FDR corrected), time coverage (d = − 0.91, p = 0.017 FDR corrected) and mean duration (d = − 0.90, p = 0.013 FDR corrected). An opposite effect was observed for MS E, which showed increased occurrence (d = 1.08, p = 0.003 FDR corrected) and time coverage (d = 1.02, p = 0.007 FDR corrected) in BPD compared to CTL.

### Exploratory Correlations Between Microstate Parameters and Spectral Power

**Fig. 5:**
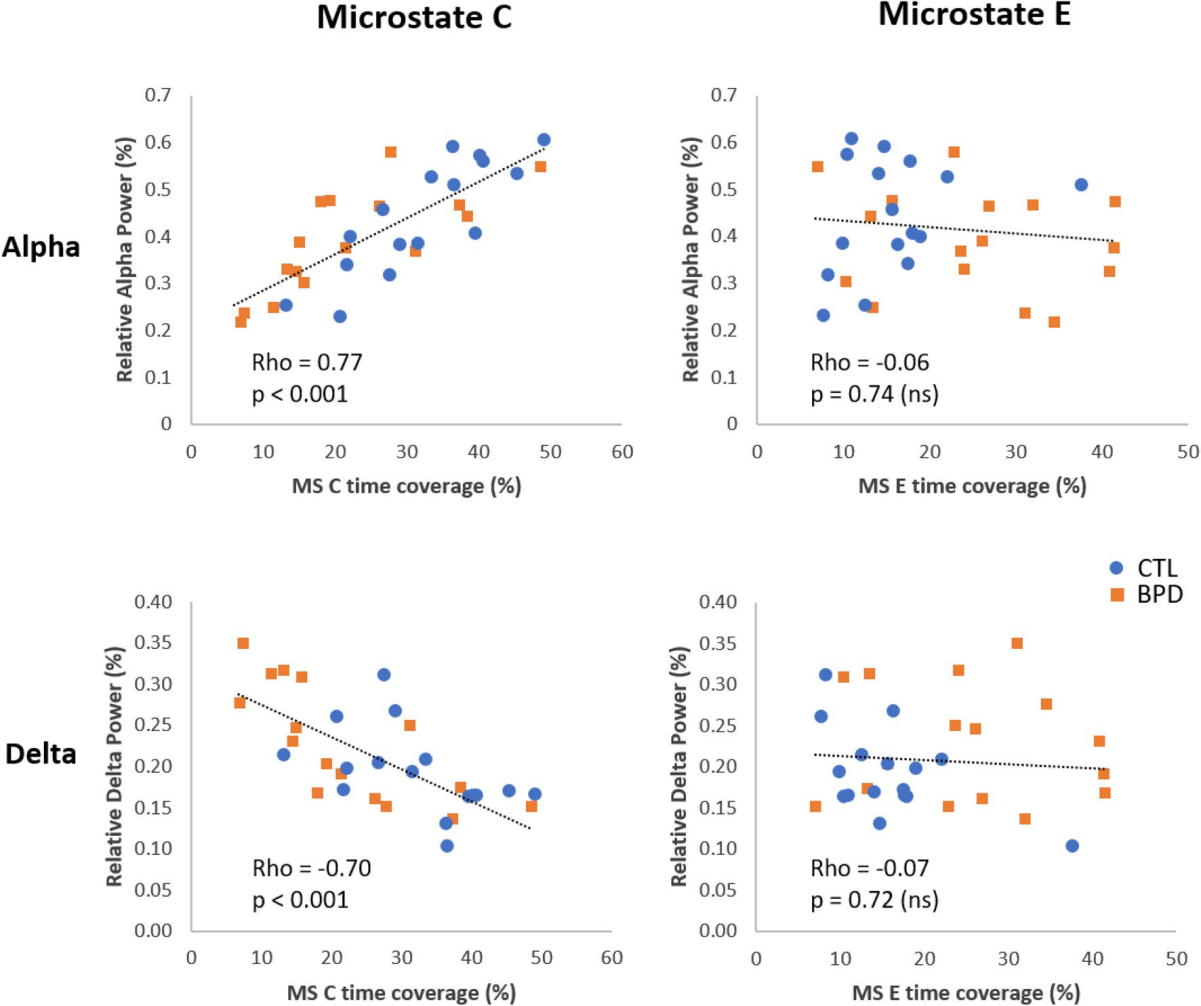
Correlations between relative alpha and delta power and MS C and E time coverage (%) in pooled CTL and BPD groups. Left column: Microstate C; right column: Microstate E. Upper row: Alpha band, lower row: Delta band. Rho: Spearman’s Rho; ns: non-significant

Here we explored the potential relationship between spectral power and the MS temporal measures that differed between the BPD and CTL groups (cf. Sect. MS segmentation). Pooling all participants, an exploratory correlation analysis (Spearman’s Rho) was performed between global relative alpha or delta power and MS C occurrence, time coverage, and mean duration, as well as MS E occurrence and time coverage. We found that global relative alpha power was positively correlated with MS C occurrence (Rho = 0.610, p < 0.001), time coverage (Rho = 0.770, p < 0.001), and mean duration (Rho = 0.804, p < 0.001). Global relative delta power was negatively correlated with MS C occurrence (Rho = − 0.546, p < 0.001), time coverage (Rho = − 0.699, p < 0.001), and mean duration (Rho = − 0.739, p < 0.001). Interestingly, no significant correlations were found for MS E measures. Figure 5 illustrates the correlations between MS C and E time coverage and global relative alpha and delta power.

### Exploratory Correlations Between Microstate Parameters and Clinical Score of Affective Lability

**Fig. 6:**
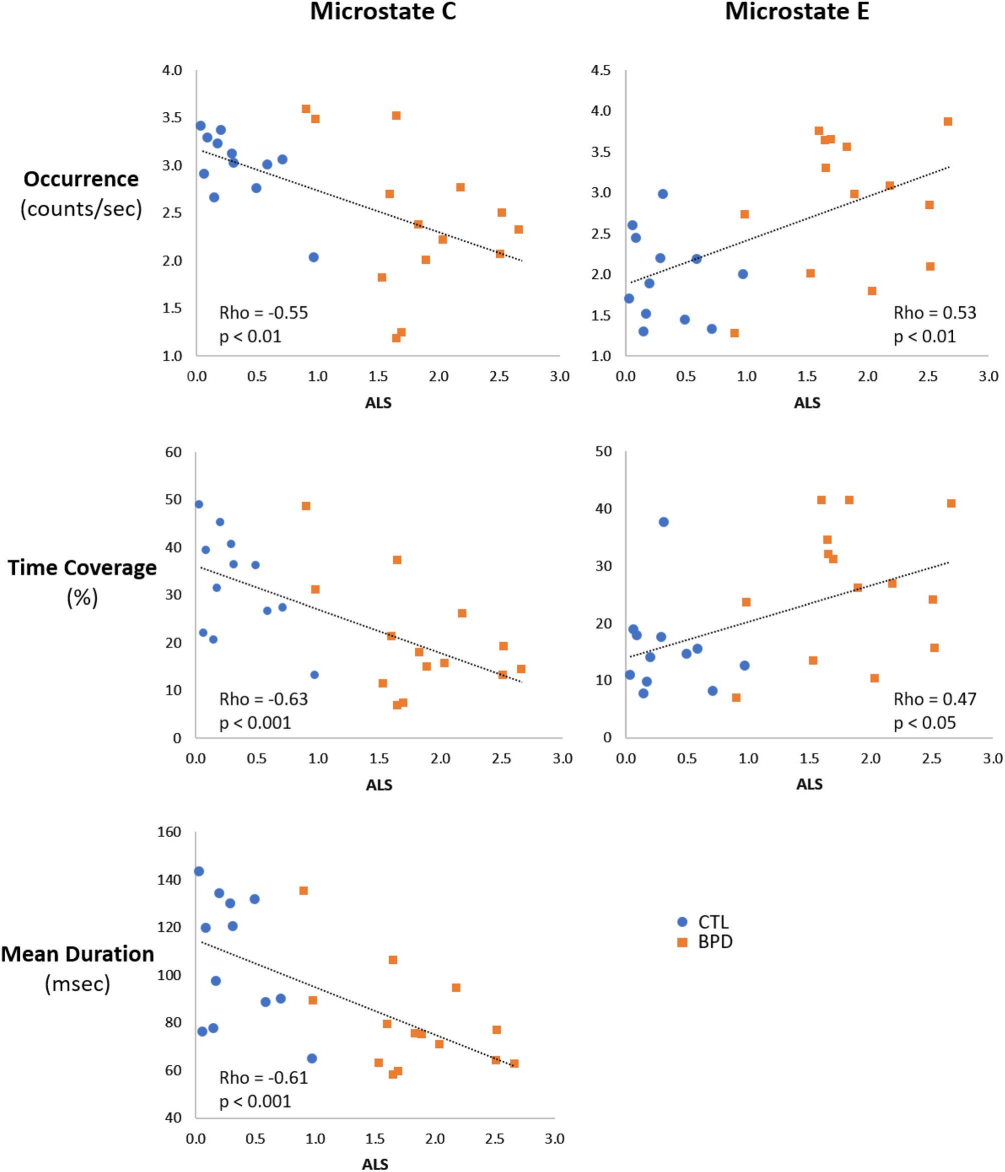
Correlations between affective lability score (ALS) and measures of MS C and E in pooled CTL and BPD groups. Left column: Microstate C, right column: Microstate E. Upper row: Occurrence, middle row: Time Coverage, lower row: Mean Duration. Rho: Spearman’s Rho

Pooling all participants, the exploratory correlation analysis (Spearman’s Rho) was performed between the ALS and the MS temporal measures that differed between the BPD and CTL groups (cf. Sect. MS segmentation). The ALS was negatively correlated with MS C occurrence (Rho = − 0.551, p < 0.01), time coverage (Rho = − 0.625, p < 0.001), and mean duration (Rho = − 0.606, p < 0.001). It was positively correlated with MS E occurrence (Rho = 0.528, p < 0.01) and time coverage (Rho = 0.469, p < 0.05). These correlations are illustrated in Fig. 6.

## Discussion

In the present study we examined two neurophysiological aspects of the resting-state EEG in BPD. Firstly, although the global relative spectral power of BPD does not differ from neurotypical subjects, exploratory univariate analyses on single channels indicated greater relative delta and lower relative alpha power in posterior regions. Secondly, we demonstrate that BPD patients share spatially equivalent MS topographies compared to neurotypical subjects—implying similar anatomical generators of EEG activity—but that their temporal dynamics are significantly altered. Compared to controls, BPD patients showed significantly reduced prevalence of MS C (for time coverage, occurrence and mean duration), together with increased prevalence of MS E (for time coverage and occurrence). Interestingly, exploratory correlation analyses revealed that increased prevalence of MS C correlated positively with global relative alpha and negatively with global relative delta power, while MS E alterations were uncoupled to changes in narrow-band spectral power.

### Power Spectrum

The global spectral power was not significantly different between the two groups in any frequency bands, indicating that global EEG power does not carry a representative hallmark of BPD. Furthermore, we did not detect alpha power asymmetry in the frontal regions, which has been suggested to be related to both motivation and emotion constructs and discussed as a potential marker of pathophysiology in BPD (Flasbeck et al. 2017). These negative findings could be the result of the limited sample size of our cohort, and therefore are to be interpreted with caution. In contrast, when running exploratory analyses on single EEG channels, we observed a reduction of posterior-midline alpha power in BPD as compared to controls. Although interpretation is limited by the risk of false positive results (i.e. absence of correction for multiple comparisons), the reduced parietal alpha power in BPD patients may suggest a state of increased cortical excitability (Mathewson et al. 2011; Romei et al. 2008), which has previously been associated with behavioral hyperarousal and/or anxiety (Barry et al. 2007; Dadashi et al. 2015). Modulation of excitability within the parietal or visual cortex is largely determined by posterior alpha activity, which mainly originates from thalamo-cortical inputs (Lorincz et al. 2008). Since the alpha band is a dominant frequency band in the generation of the MS C topography (Ferat et al. 2022), this could explain the positive relationship between MS C parameters and alpha relative power (Krylova et al. 2021; Milz et al. 2016). Conversely, the results in the delta band showed enhanced posterior-midline power in BPD. Being inversely correlated with alpha power (Newson and Thiagarajan 2019), the delta power increase also correlated negatively with MS C parameters. Overall, the lack of statistical power does not allow a firm conclusion to be made on the spectral power results, and further studies including a larger sample size will be necessary to confirm these exploratory observations.

### Microstate C

In a previous study using EEG source localization (Custo et al. 2017), MS C has been reported to be generated by the precuneus, the posterior cingulate cortex (PCC), and the left angular gyrus, all areas implicated in the default-mode network (DMN). Elsewhere, MS C presence has been associated with increases in interoceptive and self-focused thoughts (Brechet et al. 2019) and decreases in mental arithmetic tasks (Kim et al. 2021; Seitzman et al. 2017), which are also related to the activity of the “task-negative” DMN (Brechet et al. 2019; Custo et al. 2017; Michel and Koenig 2018). Hence, the reduced MS C prevalence in BPD patients suggests a reduction of task-negative brain states associated with self-processing, compatible with decreased alpha power (Chavan et al. 2013) and increased behavioral alertness (Kramer et al. 2019). Regarding other psychiatric conditions, contribution of MS C has been reported significantly reduced in eyes-closed resting EEG of adolescent ADHD (Luo et al. 2022), and unchanged in eyes-opened resting EEG of adult ADHD (Ferat et al. 2021), as compared to age-matched controls. Mirroring our findings in patients with BPD, a reduction of MS C occurrence has also been observed in a group of patients with major depressive disorder (MDD) who did not achieve remission after 8 weeks of SSRI treatment, compared to patients that achieved remission (Lei et al. 2022). Furthermore, the reduced presence of MS C was associated with poor therapeutic outcomes after treatment (Lei et al. 2022). Elsewhere, reduced MS C was also observed in mood and anxiety disorder, although this did not reach statistical significance (Al Zoubi et al. 2019).

### Microstate E

The 4 canonical classes A, B, C, D were recently complemented by the identification of 2 to 3 additional topographies, among them MS E (sometimes referred to as MS F) characterized by a topography with a posterior maximum. The anatomical generators of MS E have been related to the dorsal anterior cingulate, superior/middle frontal gyri, and insula (Brechet et al. 2019; Custo et al. 2017; Damborska et al. 2019b), which are part of the cingulo-opercular or “salience” network (CON). Damborska and colleagues (Damborska et al. 2019b) have reported on a positive correlation of MS E occurrence with intake of antidepressants, antipsychotics and mood stabilizers. They discussed a putative link between MS E and the “task-positive” CON, which plays a central role in sustaining alertness/perceptual readiness (Coste and Kleinschmidt 2016; Sadaghiani and D’Esposito 2015), and whose disrupted connectivity was observed in depression (Wu et al. 2016). A complementary view relates the CON with the integration of autonomic processes, by which it is engaged in assessing the salience of internal and external stimuli (Seeley 2019; Seeley et al. 2007). Increased prevalence of MS E in BPD patients is thus compatible with both a higher sustained alertness and an exacerbated response of the CON in relation with salience processing (Sadaghiani and D’Esposito 2015). Moreover, the CON partly overlaps with the fronto-limbic emotion regulation network, whose impairment might mediate emotional dysregulation in BPD (Krause-Utz et al. 2014; O’Neill and Frodl 2012). Recent findings from our own group have identified a positive link between BOLD signal variability in the fronto-limbic network and the severity of emotional dysregulation in BPD (Kebets et al. 2021), that could explain the association between the observed abnormalities in MS E and emotional instability in BPD. In contradistinction to our results in BPD patients, reduced presence of MS E has been found in post-traumatic stress disorder (PTSD) (Terpou et al. 2022) as well as in MDD, where it also negatively correlated with depression inventory scores (Qin et al. 2022). Hence increased prevalence of MS E may correspond to a unique neuromarker of BPD, that is independent from microstate signatures of patients with PTSD or MDD.

### Association of MS C and E with Emotional Instability

We further explored the potential associations between EEG microstate abnormalities and the ALS, a widely employed index of emotional dysregulation severity in BPD. To overcome the issue of small sample size in our BPD group, we opted for pooling the two groups for these analyses. The interpretation of the results is complicated by the possibility that the data derive from separate distributions (Simpson’s paradox, Simpson 1951). However, if one assumes the pooled data come from the same sampling distribution (i.e. patients are on the same continuum as controls), then these correlations underline a potentially significant relationship between emotional dysregulation and microstate measures. MS C and MS E showed opposite behavior in their relationship with ALS, consistent with their abnormality in BPD. MS C, significantly reduced in BPD, was negatively correlated with ALS, while MS E, significantly increased in BPD, was positively correlated with ALS. These observations suggest the relevance of the MS C and E measures as potential neuromarkers of emotional instability in BPD. Further investigations are needed to establish a possible mediating link between these spatiotemporal indices of brain activity and emotional impairment in BPD.

### What EEG Resting-State Analyses Tell Us About BPD Neurophysiology

The results based on microstates are more statistically significant and conclusive than those based on spectral power, suggesting a greater sensitivity of EEG spatiotemporal measures in BPD. The finding of significant exploratory correlations between one microstate class (i.e. MS C) and alpha power on the one hand, and delta on the other, indicates that both of these measures may be associated with an overlapping aspect of the disorder. However, as this was not the case for microstate E, the latter could represent a valuable, additional neuromarker of the disorder, referring more particularly to emotional instability. The reduced posterior-midline alpha power in BPD patients compared to neurotypical subjects suggests increased cortical hyperexcitability in this disorder. This finding is in line with recent EEG evidence that BPD patients at rest demonstrate higher stages of vigilance compared to controls (Kramer et al. 2019). This could potentially be also linked to elevated noradrenergic activity in the locus coeruleus reported in BPD (Kramer et al. 2019), in turn responsible for various effects associated with stress, including elevated arousal and deficiencies in attentional and cognitive functions (Berridge and Spencer 2017). Further analyses on larger samples of patients would be necessary to confirm the link between microstate E and clinical scores, in particular emotional lability.

### Limitations

The present study has some limitations. First, the sample size of each group is quite small, limiting statistical power. Second, the patient sample was almost all female, which could potentially be a source of bias. Even if in epidemiological studies the gender ratio between male and female is near to 1, usually in clinical settings and thus in research coming from these facilities more than 75% of people diagnosed with BPD are women (Sansone and Sansone 2011; Skodol and Bender 2003). The reasons for this significant overrepresentation of women are still unknown; there may be biological, psychosocial, cultural and psychological factors that predispose women with BPD to more often seek psychological/psychiatric help. Our own data simply reflect this worldwide overrepresentation of women in specialized units. Although comprising more male subjects, the gender of our age-matched control group did not differ significantly from the patient group.

### Conclusions

To our knowledge, the current study is the first to analyze the resting-state dynamics of EEG microstates in patients with BPD, where we observed a reduced prevalence of MS C and an increased prevalence of MS E. These findings, together with the exploratory observations of reduced (enhanced) power in the alpha (delta) bands, are compatible with a signature of cortical hyperactivation in BPD patients, associated with a state of elevated cortical arousal and/or behavioral vigilance. Although the present findings need replication in larger samples, the application of MS analysis appears to offer more specific neuromarker(s) of BPD pathophysiology and diagnosis, when compared to classical analyses using spectral power. This approach could also hold promise for developing future neuromarker-based treatments (e.g. EEG neurofeedback).

### Supplementary Information

Below is the link to the electronic supplementary material.

Supplementary material 1 (CSV 17.2 kb)
